# The Effect of IL-4 Gene Polymorphisms on Cytokine Production in Patients with Chronic Periodontitis and in Healthy Controls

**DOI:** 10.1155/2014/185757

**Published:** 2014-10-29

**Authors:** Jirina Bartova, Petra Borilova Linhartova, Stepan Podzimek, Tatjana Janatova, Kazi Svobodova, Antonin Fassmann, Jana Duskova, Jaromir Belacek, Lydie Izakovicova Holla

**Affiliations:** ^1^Institute of Clinical and Experimental Dental Medicine, General University Hospital and First Faculty of Medicine, Charles University, Karlovo nam. 554/32, 12808 Prague, Czech Republic; ^2^Department of Pathophysiology, Faculty of Medicine, Masaryk University, 65691 Brno, Czech Republic; ^3^Clinic of Stomatology, Faculty of Medicine, Masaryk University and St. Anne's University Hospital, 65691 Brno, Czech Republic; ^4^Institute of Biophysics and Informatics, First Faculty of Medicine, Charles University, 12108 Prague, Czech Republic

## Abstract

Chronic periodontitis (CP) is an inflammatory disease of the teeth-supporting tissues in which genetic predisposition, dental plaque bacteria, and immune mechanisms all play important roles. The aim of this study was to evaluate the occurrence of IL-4 gene polymorphisms in chronic periodontitis and to investigate the association between polymorphisms and cytokines production after bacterial stimulation. Sixty-two subjects (47 CP patients and 15 healthy controls) with detected two polymorphisms in the IL-4 gene (-590C/T and intron 3 VNTR) were examined. Production of cytokines (IL-1*α*, IL-1*β*, IL-4, IL-5, IL-6, IL-10, IL-17, TNF*α*, INF*γ*, and VEGF) was studied after *in vitro* stimulation of isolated peripheral blood by mitogens (Pokeweed mitogen, Concanavalin A), dental plaque bacteria (*Aggregatibacter actinomycetemcomitans, Tannerella forsythia, Porphyromonas gingivalis, and Prevotella intermedia*), and Heat Shock Protein (HSP) 70 by the Luminex* multiplex cytokine analysis *system. The results were correlated with IL-4 genotypes in patients with CP and healthy controls. The mononuclear cells isolated from peripheral blood of CP patients with selected IL-4 polymorphisms significantly altered the production of IFN*γ*, IL-10, IL-1*β*, IL-1*α*, TNF*α*, and IL-6 after stimulation by HSP 70 or selected bacteria (from *P* < 0.001 to *P* < 0.05). IL-4 gene polymorphisms may influence the function of mononuclear cells to produce not only interleukin-4 but also other cytokines, especially in patients with CP.

## 1. Introduction

Chronic periodontitis (CP) is an inflammatory disease of the teeth-supporting tissues in which genetic predisposition, dental plaque bacteria, and immune mechanisms all play important roles. Bacteria which are involved in the pathogenesis of CP include* Porphyromonas gingivalis*,* Aggregatibacter actinomycetemcomitans, Tannerella forsythia, Prevotella intermedia,* and* Fusobacterium nucleatum*. The participation of these bacteria in the pathogenesis of CP correlates with an increased level of specific antibodies in sera [1]. Suspect pathogens isolated from dental plaque continually stimulate cells in the periodontal tissue to produce cytokines as well as other biologically active molecules. The development and regulation of the immune response depend on the local production and quantities of cytokines and other mediators which influence disease progression or resolution. The disease progression depends on the increased production of proinflammatory cytokines (IL-1*α*, IL-1*β*, IL-6, IL-8, and TNF*α*), metalloproteinases, and prostaglandins or decreased production of anti-inflammatory cytokines (IL-10, TGF*β*) and inhibitors of metalloproteinases [2].

Based on the concept of different functions of TH1 and TH2 lymphocytes [3], Seymour et al. [4] proposed the hypothesis that, in patients with progressing CP, clones of TH2 are generated upon activation with bacteria, while, in the nonprogressing disease, stable lesions are regulated by TH1 clones. We confirmed these findings in our previous longitudinal studies in which we followed, for a period of 10 years, patients with early onset periodontitis (EOP, now known as aggressive periodontitis) with changes in periodontal tissues diagnosed between the ages of 17 and 25. Such patients repeatedly exhibited increased production of IL-4 (TH2) upon stimulation of peripheral blood lymphocytes with dental plaque bacteria while their healthy siblings and other genetically unrelated controls with intact periodontal tissues produced a significantly increased level of IFN*γ* (TH1) [5]. As mentioned above, both the onset and progression of periodontal diseases are strongly influenced by the genetic predisposition of the individual. The heritability of the disease varies from a nearly 100% share in “Mendelian/syndromological” forms to states with a low proportion of heritability and a high effect of environmental factors. Rateitschak et al. [6] described the genetic predisposition for early onset periodontitis; according to Michalowicz [7], genes are responsible for more than 50% of the risk of chronic periodontitis. The 1990s witnessed the beginning of the phase of genetic analysis at the level of so-called “candidate” genes, that is, genes mostly coding proinflammatory cytokines, chemokines, metalloproteinases, and other factors associated with the production of these mediators and their presumed role in the pathogenesis of the disease [8, 9]. The current development of methodological options underlies the present era of genome-wide studies [10]. Polymorphisms in certain alleles of cytokines have been associated with susceptibility to a wide range of infectious or immune diseases, including periodontitis [11–16] and others. The human interleukin-4 (IL-4) gene is mapped within the cytokine gene cluster on chromosome 5q31-33 and contains several polymorphisms; some of them are implicated in the regulation of IL-4 production. Recently, these polymorphisms have attracted widespread attention, especially the IL-4 gene promoter -590C/T (rs2243250) polymorphism and a 70 bp variable number of tandem repeat (VNTR) polymorphism in its third intron. It was proposed that an increased responsiveness of the -590C/T allele and the (70 base pairs (bp)) 2 repeat allele to transcriptional activation might lead to overexpression of IL-4 [17].

However, the role of these polymorphisms in cytokine genes in the production of cytokines in response to stimulation by dental plaque bacteria in periodontal disease has not yet been studied. The aim of this study was to assess the influence of IL-4 (-590C/T) and IL-4 VNTR (variable number of tandem repetitions in intron 3 gene) polymorphisms on cytokine production after stimulation of isolated peripheral blood mononuclear cells by dental plaque bacteria, mitogens, and HSP70 in patients with periodontitis and in healthy controls.

## 2. Materials and Methods

### 2.1. Study Population

All patients with chronic periodontitis (CP, *N* = 47) were recruited from the patient pool of the Periodontology Department, Clinic of Stomatology, St. Anne's Faculty Hospital Brno, from 2010 to 2012. Inclusion criteria were good general health, diagnosis of generalized chronic periodontitis according to the International Workshop for a Classification of Periodontal Diseases and Conditions for Chronic Periodontitis [18], and agreement with sample collection for genetic/immunological examinations. Exclusion criteria included history of cardiovascular disorders (such as coronary artery diseases or hypertension), diabetes mellitus, malignant diseases, immunodeficiency, current pregnancy or lactation, and smoking. The control group (healthy/nonperiodontitis subjects, *N* = 15) were selected randomly during the same period as patients and matched for age, gender, and nonsmoking status. All controls had at least 20 remaining teeth, were general healthy, and agree with genetic/immunological examinations. Exclusion criteria were the same as those applied with patients with periodontitis.

Diagnosis of nonperiodontitis/periodontitis was based on a detailed clinical examination, medical and dental history, tooth mobility, and radiographic assessment. Probing depth (PD) and clinical attachment loss (CAL) were collected by UNC-15 periodontal probe from six sites on every tooth present. All patients had to have at least three teeth with bleeding on probing (BOP), PD of >4 mm, and CAL of ≥3 mm in all quadrants (excluding the third molars); however, they were treated patients without active phase of disease. The loss of alveolar bone was determined radiographically. We used the Mühlemann index to evaluate decreases in alveolar bone levels [19]. The control group (healthy periodontium) consisted of subjects with no history or clinical signs of gingivitis and/or periodontitis (no PPD of >4 mm, no loss of clinical attachment around any tooth, and no radiographic sign of bone resorption).

The study was performed with the approval of the Committees for Ethics of the Medical Faculty, Masaryk University Brno and St. Anne's Faculty Hospital. Written informed consent was obtained from all participants in line with the Helsinki declaration before inclusion in the study.

### 2.2. Isolation of Genomic DNA

DNA for genetic analysis was extracted from the peripheral blood leukocytes (5 mL) using standard phenol/chloroform procedures with proteinase K according to Sambrook et al. [20].

### 2.3. Genetic Analysis

The GenBank accession number was AF395008 for the reference genomic sequence used for IL-4 alignments. The IL-4 SNP polymorphism at position -590C/T (rs2243250) was genotyped by PCR-RFLP analysis according to previously published methods [21] with slight modification. DNA was amplified with the following primers: [5′-ACT AGG CCT CAC CTG ATA CG-3′ (sense), 5′-AGG TGT CGA TTT GCA GTG AC-3′ (antisense)] as a product with 646 bp length. PCR was carried out in a volume of 15 *μ*L containing 50 ng of genomic DNA, 0.3 *μ*M of each primer, 0.5 U of DNA polymerase (Biotools B&M Labs S. A., Madrid, Spain), 5 mM of MgCl_2_, 10x reaction buffer MgCl_2_ free (Biotools B&M Labs S. A., Madrid, Spain), and 0.5 mM of deoxyribonucleoside triphosphate mix (Roche, Basel, Switzerland). Denaturation for 5 min at 95°C was followed by 35 cycles of 95°C for 45 s, 56°C for 45 s, and 72°C for 1 min. The last synthesis step was extended to 10 min at 72°C. The PCR products were then digested with BsmFI restriction enzyme and separated on 3% agarose gel. The restriction was performed in a volume of 12 *μ*L containing 7 *μ*L of the PCR product, 10x NeBuffer 4, and 4 U of BsmFI enzyme (New England Biolabs, Hitchin, UK) and incubated for 4 hours at 65°C. Genotypes were described as CC (601 + 45 bp), CT (646 + 601 + 45 bp), and TT (646 bp).

VNTR (IL-4 70-bp repeat) polymorphism in intron 3 of the IL-4 gene was detected by a modification of the PCR method described by Mout et al. [22]. DNA was amplified with the following primers: [5′-TAG GCT GAA AGG GGG AAA GC-3′ (sense), 5′-CTG TTC ACC TCA ACT GCT CC-3′ (antisense)]. PCR was carried out in a volume of 15 *μ*L containing 100 ng of genomic DNA, 0.3 *μ*M of each primer, 0.5 U of DNA polymerase (Biotools B&M Labs S.A., Madrid, Spain), 3.5 mM of MgCl_2_, 10x reaction buffer MgCl_2_ free (Biotools B&M Labs S.A., Madrid, Spain), and 0.5 mM of deoxyribonucleoside triphosphate mix (Roche, Basel, Switzerland). Denaturation for 5 min at 95°C was followed by 35 cycles of 95°C for 45 s, 56°C for 45 s, and 72°C for 1 min. The last synthesis step was extended to 10 min at 72°C. Amplified products were separated on 2% agarose gel; the size of the product corresponds to the number of the repeats present (allele 1 = 3 repeats (254 bp); allele 2 = 2 repeats (184 bp)). The rarely occurring allele type 3 with only a single repeat (114 bp) was not present in our set.

The genotyping was performed by P. B. L. unaware of the phenotype. DNA samples with known genotypes, which were identified by sequencing of these samples in the previous study by Holla et al. [14], were used as positive controls for both polymorphisms.

### 2.4. Cultivation of Dental Plaque Bacteria

Samples of the sulcular fluid for the cultivation of bacteria (*A. actinomycetemcomitans, P. gingivalis, T. forsythia, *and* P. intermedia*) were collected from sites of periodontium with the worst affected areas of periodontium. A volume of 50 *μ*L of a sample diluted by 2 mL Brain Heart medium was inoculated on the surface of microbiological diagnostic media in Petri dishes. Selective cultivation of bacteria was performed according to the method of Slots [23] on TSBV agar incubated at 37°C in 10% CO_2_ atmosphere created by GasPak CO_2_ bags (Oxoid) in an anaerobic pot. Bacteria colonies were harvested after 5 to 7 days and bacterial suspension containing 10^10^ cells/mL was inactivated by incubation at 120°C for 20 minutes. Samples were centrifuged for 10 minutes at 600 g. Sediments were diluted with PBS and centrifuged for 10 minutes at 600 g and then washed 3 times. The washed sediments were diluted by X-Vivo medium to optical density corresponding to 10^10^ cells/mL.

### 2.5. Immunological Examination

20 mL of peripheral blood was collected from the subjects under examination to BD Vacutainer with sodium heparin as anticoagulant (BD, Plymouth, UK). Peripheral blood mononuclear cells (PBMC) were isolated from heparinized blood by Histopaque 1.077 (Sigma, St. Louis, MO, USA) gradient centrifugation for 30 min at 600 g. The white layer in the interphase containing mononuclear cells was collected and washed with X-vivo 10 medium at 400 g and 200 g and then diluted with X-Vivo 10 serum-free tissue medium (Cambrex) to the concentration of 10^7^ cells/mL. 100 *μ*L cells were cultivated for 3 days at 37°C in 5% CO_2_ atmosphere and stimulated with 100 *μ*L of the bacteria with concentration of 10^9^/mL (*A. actinomycetemcomitans, P. gingivalis, T. forsythia,* and* P. intermedia*), HSP 70 (Sigma, 5 *μ*g/mL), Pokeweed mitogen (PWM, Sigma, 2.5 *μ*g/mL), and Pokeweed mitogen in costimulation with Concanavalin A (PWM, Sigma, 2.5 *μ*g/mL + ConA, Sigma, 10 *μ*g/mL) and supplemented with X-vivo 10 medium to l mL (Table 2). After the 3 days of cultivation, the medium was collected and stored at −20°C for the determination of cytokines (IL-1*α*, IL-1*β*, IL-4, IL-5, IL-6, IL-10, IL-17, IFN*γ*, TNF*α*, and VEGF) by the Luminex multiplex method (LUMINEX 100TM analyzer, R&D Systems, USA). Cytokine production in subjects with different genotypes of IL-4 polymorphism was then compared.

### 2.6. Statistical Analysis

Statistically significant results (for *P* < 0.05, *P* < 0.01, and *P* < 0.001) are indicated in Tables 3(a) and 3(b) representing medians for dichotomous variants, separately for patients with periodontitis (*N* = 47) and for healthy controls (*N* = 15). Calculations were performed in SPSS (version 17.0).

**Figure 1:**
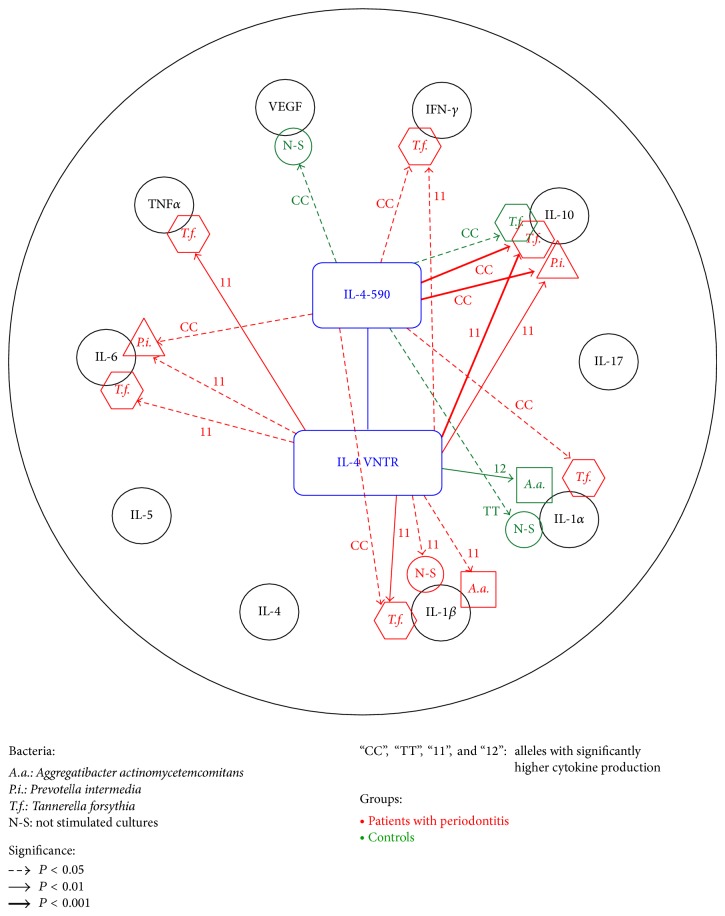
Statistically significant (higher) production of cytokines (unstimulated and stimulated by bacteria) on alleles of IL-4 SNP genes for patients with periodontitis (*N* = 47) and healthy controls (*N* = 15), evaluation by Spearman rank correlations. Remark: our application of Spearman correlation analysis gives us the very similar results in comparison with levels of significance observed by K-W method with preliminary aggregation of two adjacent minority alleles (see legend under Table 3); application of Spearman rank correlations does not reduce any data and slightly more supports the hypotheses related to expression of cytokines on homozygous allele.

The differences in production of cytokines inside the two groups of patients (with periodontitis and controls) in combination with investigated IL-4 polymorphisms were preliminarily analyzed by multiple use of the Kruskal-Wallis test [24] with two adjacent alleles with minority representations in sample populations being always tied into one (see Tables 3(a) and 3(b) where alleles with significantly higher levels of cytokines are marked right over sample medians). Alternatively, we computed two matrices of Spearman rank correlation coefficients [25] (one for patients with periodontitis and another one for controls) between observed levels of cytokines and IL-4 polymorphisms and compared the observed levels of significance for the two methods (see Figure 1, where only statistically significant relations are highlighted by arrows.) Statistically significant results (for *P* < 0.05, *P* < 0.01, and *P* < 0.001) are indicated in Tables 3(a) and 3(b) and Figure 1.

## 3. Results

The mean ages for periodontitis patients (51.53 ± 6.52 years, mean ± SD) and healthy subjects (46.13 ± 9.65) did not differ between the two groups. There were no significant differences (*P* > 0.05) between subjects with periodontitis and controls regarding the average age, nonsmoking status, and ratio of males/females (4/11 in controls, 21/26 in patients with periodontitis) (Table 1).

Both studied polymorphisms (IL-4 -590C/T and VNTR in intron 3) were in the Hardy-Weinberg equilibrium in the control group. Allele and genotype frequencies of IL-4 polymorphisms were not significantly different between the subjects with periodontitis and controls (*P* > 0.05; data not shown). Among 45 patients with CP, there were 2 patients with slight (1 or 2 mm CAL), 11 patients with moderate (3 or 4 mm CAL), and 32 patients with severe form of CP (≥5 mm CAL). When comparing genotypes (IL-4 -590CC versus CT + TT and IL-4 VNTR 11 versus 12 + 22) between patients with the slight/moderate (*N* = 13) and severe (*N* = 32) form of periodontitis, we found no statistically significant differences between both groups (*P* = 0.50 for IL-4 -590C/T and *P* = 0.61 for IL-4 VNTR polymorphisms).

Both patients and healthy controls were divided into groups according to the polymorphisms in IL-4 gene (homozygote for CC versus CT + TT genotypes for -590C/T variant and homozygote 11 versus 12 + 22 genotypes for VNTR). Tables 3(a) and 3(b) illustrate medians of the cytokine levels measured after a 3-day* in vitro* stimulation of lymphocytes isolated from peripheral blood of patients and healthy controls by dental plaque bacteria, mitogens, and HSP70.

The results in Table 3(b) show that the measured values of IL-4 and IL-5 (in pg/mL) were very low and no significant differences between groups in the levels of these cytokines were detected. Conversely, the level of IL-6 was relatively high in both patients with periodontitis and healthy controls. The levels of IL-6 after stimulation of lymphocytes by the bacterium* P. intermedia* in patients carrying the IL-4 -590CC and IL-4 VNTR 11 genotype were significantly higher than in the carriers of -590CT genotypes and IL-4 VNTR 12 + 22 (*P* < 0.05). A similar statistically significant difference in the production of IL-6 and also TNF*α* after stimulation by* T. forsythia* was detected in the carriers of IL-4 VNTR 11 genotype when compared with patients carrying genotypes 12 or 22 (*P* < 0.05). The results in Table 3(a) show that lymphocytes from patients with periodontitis carrying CC genotype of the IL-4 -590 polymorphism or 11 genotype of the IL-4 VNTR produced more IFN*γ* (*P* < 0.01 and *P* < 0.05, resp.) after stimulation with* T. forsythia* and also more IL-10 after stimulation with* T. forsythia* and* P. intermedia* (from *P* < 0.001 to *P* < 0.05). IL-10 was significantly increased in group of healthy persons carrying genotypes CC IL-4 -590 after stimulation with* T. forsythia*. On the other hand, no significant differences in the production of IL-17 and IL-1*α* after stimulation by dental plaque bacteria were determined in periodontitis patients carrying the IL-4 gene polymorphisms in this study. Production of IL-1*β* was significantly higher in patients with periodontitis carrying the CC genotype IL-4 -590C/T variant after stimulation with* T. forsythia *and in the unstimulated culture (*P* < 0.05). Subjects carrying genotype IL-4 VNTR 11 were found to have a higher production of IL-1*β* after stimulation with nearly all agents (from *P* < 0.01 to *P* < 0.05) except for the stimulation with* P. gingivalis* and* P. intermedia* (*P* > 0.05).

Within the group of healthy controls, statistically significant differences were observed only between subjects with CC versus CT and TT genotypes in IL-4 -590 polymorphism after stimulation with* T. forsythia* (*P* < 0.05). The highest difference in the production of cytokine IL-1*α* was observed between controls carrying genotypes CT and TT versus CC after stimulation with HSP70 and* A. actinomycetemcomitans* and in unstimulated culture (*P* < 0.05). A significantly higher production of IL-1*α* was found only in the unstimulated culture in patients with genotypes IL-4 VNTR 11. We did not find any significant differences in cytokines production after stimulation by PWM, PWM + Con A, and HSP70 in both examined groups.

Figure 1 shows the results of a parallel evaluation of these relationships by Spearman's correlation analysis; they are identical with the data in Tables 3(a) and 3(b) and clearly show the relationship of both IL-4 polymorphisms to cytokine production after the stimulation by the studied bacteria. Figure 1 shows that, in patients with periodontitis, homozygotes “CC” of IL-4 -590C/T polymorphism and “11” of IL-4 VNTR play an important role in the response of peripheral blood lymphocytes to stimulation with bacteria.* T. forsythia* is an important stimulator of production of cytokines TNF*α*, IL-6, IL-10, IFN*γ*, IL-10, and IL-1*β* while* P. intermedia* affects the* in vitro* production of IL-6 and IL-10 in patients with periodontitis. Production of IL-17, IL-4, and IL-5 was not affected by the short-term stimulation by any of the above factors (Figure 1).

## 4. Discussion

The interactions between periodontal pathogens and the immune response of the organism require knowledge of the pathogenesis of periodontal disease. The activation of dental plaque bacteria involves the development of local and systemic humoral and cellular immunity. The illness is characterized by accumulation of lymphocytes in the periodontal tissues; the inflammatory infiltrate is dominated at the beginning by T lymphocytes while the later chronic phase shows the shift to B lymphocytes and plasma cells [26].

After* in vitro* stimulation with* P. gingivalis* and* F. nucleatum*, most lymphocytes obtained from biopsies of periodontitis-affected tissues were of the TH2 phenotype [4]. Inactivation of IFN-*γ* and IL-2 by gingipains from* P. gingivalis* at inflammatory sites could also downregulate TH1 responses (associated with nonaggressive periodontal lesions) and promote TH2 pathways and polyclonal B-cell activation in advanced periodontitis [27]. Based on our previous experience and discussed publications, we have selected periopathogens and mitogens to be tested in this study. PWM was used as T and B cells stimulator and it was used in combination with Concanavalin A that activates T regulatory mechanisms; HSP70 was used as an indicator of shock response of inflamed tissue.

We suppose low levels of investigated cytokines after stimulation with* P. gingivalis* were found as a consequence of short time stimulation or by inactivation of investigated cytokines by gingipains.

The results of the present study point to the importance of single nucleotide IL-4 gene polymorphisms in periodontal disease. One of the most important findings is the proof that the -590C/T polymorphism in the promoter region and VNTR in intron 3 of the gene encoding IL-4 affect not only the production of this interleukin, as described earlier [28–30], but also the production of other cytokines in response to the action of dental plaque bacteria or selected mitogens, this effect being much more conspicuous in patients with chronic periodontitis when compared with healthy controls.

The greatest effect of stimulation with* T. forsythia* on production of IL-10 was observed in patients with periodontitis carrying the genotypes CC (-590C/T) and 11 (VNTR) of the gene encoding IL-4 while the increase in the levels of IFN*γ*, IL-1 *β*, IL-6, and TNF*α* in patients with the same genotypes was lower (*P* < 0.05). The levels of regulatory cytokine IL-10 in patients with periodontitis carrying the appropriate genotypes of IL-4 polymorphisms were also significantly increased, after stimulation with* P. intermedia*. In contrast to patients that exhibited increased production of the abovementioned cytokines in the presence of genotypes CC or 11, higher production of IL-1*α* after stimulation with HSP70 and the bacterium* A. actinomycetemcomitans* was found in control subjects carrying the combination CT + TT (IL-4 -590C/T) and 12 (IL-4 VNTR).

Based on our previous results [5, 15], we assumed that, after stimulation with dental plaque bacteria, IL-4 gene polymorphisms in periodontitis patients would in particular affect the production of cytokine IL-4. Contrary to this assumption, no significant increase was found in the level of IL-4, while stimulation with* T. forsythia* and* P. intermedia* resulted in significantly higher levels of other cytokines. These results are in keeping with the data in a number of recent studies which documented that polymorphisms in genes encoding some cytokines or their receptors can affect the production of not only their own but also other mediators. The results of the study by Wallis et al. [31] indicate that I50V SNP in part IL-4R controls production of IL-17 by TH17 cells cultured from healthy individuals. Similarly, the minor allele of the IL-4R SNP (rs1805010), which confers impaired IL-4 signaling and has been associated with an aggressive destructive course of rheumatoid arthritis (RA), contributes to increased TH17 cell frequency, enhanced clinical activity, and accelerated radiographic progression in RA by rendering CD4 T cells from RA [32].

Cytokines play a key role in modulation of immune reaction. All cytokines are pleiotropic. They have the ability to interact with specific receptors on the cell surface and can modulate receptor expression for other cytokines. The same biological function of cytokines is influenced by several distinct cytokines (cytokine cross talk). This is a biologically important feature: if one cytokine is absent or its level limited, substitution by another cytokine exists and may be regulated by IL-4 gene polymorphisms in patients with CP [33]. Our results presented in Table 3(b) show that IL-4 gene polymorphisms influence the production of another TH2 cytokine IL-6. IL-6 is an interleukin that acts as both a proinflammatory and anti-inflammatory cytokine. The role of IL-6 is anti-inflammatory: it has an inhibitory effect on TNF*α* and IL-1 production and it activates production of IL-1 ra and regulatory IL-10. We suppose that the higher median detected in the healthy group as compared to periodontitis patients group after stimulation of their lymphocytes by* P. intermedia* may be explained by the anti-inflammatory properties of IL-6.

There were several limitations to this study. For instance, the short cultivation time of lymphocytes isolated from peripheral blood with bacteria and mitogens. Also, the healthy control and periodontitis groups were not precisely matched by sex. Lastly, the small sample size precludes any robust claims and points to the need for additional large longitudinal studies of cohorts of patients with periodontitis. For this reason, two statistical methods for evaluation of results were used.

It appears that the knowledge of the genetic background of chronic periodontitis will remain for quite some time on the level of knowledge of individual “potential” risk or protective factors whose effect on the disease will depend on a broader, still unexplored genetic background and a number of epigenetic factors. The differences in the findings published in individual studies are hindered by the fact that individual populations in the world can differ in the frequencies of the alleles of appropriate polymorphisms. Other factors that may play a role are different approaches to data evaluation and differences in clinical evaluation and selection of patients. For example, the periodontitis of young subjects, formerly presented as early onset periodontitis or juvenile periodontitis, is currently classified as aggressive periodontitis. In a longitudinal (10-year) study, we explored the progression of early onset periodontitis in patients aged 17–25 years at the beginning of the study. Over the years, the disease in some patients progressed while in others it did not [15, 34]. According to current criteria, all patients would be classified at the beginning of the study as suffering from aggressive periodontitis. This fact points to the importance of preventive examinations focused on the initial changes of the periodontium of young subjects and on early preventive therapy.

## 5. Conclusion

Our study suggests that polymorphisms in the IL-4 gene not only affect the production of the IL-4 cytokine but can influence production of several other cytokines, such as IL-10, IFN*γ*, IL-1*β*, IL-6, and TNF*α*, which in turn can affect periodontal disease. Further studies are needed to clarify the association of these polymorphisms with cytokine production in other populations.

## Figures and Tables

**Table 1:** Demographic and clinical data in patients with chronic periodontitis (CP) and nonperiodontitis controls.

	Patients with CP	Controls

Number of subjects	47	15
Mean age (yrs ± SD)	51.53 ± 6.52	46.13 ± 9.65
Age range (yrs)	From 35 to 65	From 35 to 66
Sex (M/F)	21/26	4/11
PD (mm; mean ± SD)	4.9 ± 1.5^*^	1.9 ± 0.6
CAL (mm; mean ± SD)	5.4 ± 2.1^*^	2.1 ± 0.7

^*^ *P* < 0.05.

**Table 2:** Scheme of *in vitro* stimulation of mononuclear cells.

	Cells (10^7^ cells/mL)	Stimulation	Cultivation X-Vivo medium
PWM	100 *µ*L	100 *µ*L PWM	800 *µ*L
PWM + ConA	100 *µ*L	100 *µ*L PWM + 100 *µ*L ConA	700 *µ*L
HSP70	100 *µ*L	100 *µ*L	800 *µ*L
*A.a. *	100 *µ*L	100 *µ*L *A.a. *	800 *µ*L
*P.g. *	100 *µ*L	100 *µ*L *P.g. *	800 *µ*L
*T.f. *	100 *µ*L	100 *µ*L *T.f. *	800 *µ*L
Unstim.	100 *µ*L		900 *µ*L

PWM: Pokeweed mitogen, ConA: Concanavalin A, HSP70: Heat Shock Protein 70, *A.a*.: *Aggregatibacter actinomycetemcomitans*, *P.g.*: *Porphyromonas gingivalis*, *T.f.*: *Tannerella forsythia*, and unstim.: without stimulation.

**(a):** 

Locus	Periodontitis patients								Healthy controls							
	IL-4-590				IL-4VNTR				IL-4-590				IL-4VNTR			
Polymorphism	CC		CT + TT		11		12 + 22		CC		CT + TT		11		12 + 22	
(Number of patients)	(30)		(16 + 1)		(28)		(17 + 1)		(8)		(5 + 2)		(10)		(5 + 0)	
Cytokines [pg/mL]	Median	(Min/max)	Median	(Min/max)	Median	(Min/max)	Median	(Min/max)	Median	(Min/max)	median	(Min/max)	Median	(Min/max)	Median	(Min/max)
IFNg_PWM	116.7	(0/7816.5)	180.6	(0.8/3898.5)	116.8	(0.2/7816.5)	147.6	(0/3898.5)	4.5	(0.5/983.9)	178.6	(2.2/1073.7)	4.5	(0.5/983.9)	178.6	(2.2/1073.7)
IFNg_P + C	92.8	(0.3/7215)	71.2	(0.3/1280.4)	113.6	(0.6/7215)	56.0	(0.3/1280.4)	126.9	(1.1/1009.9)	81.6	(2/1081)	126.9	(1.1/1009.9)	81.6	(2/1081)
IFNg_HSP70	1.5	(0/7282)	0.3	(0/781.9)	1.1	(0/7282)	0.3	(0/781.9)	1.1	(0.1/550.5)	0.3	(0.2/552.4)	1.1	(0.1/550.5)	0.3	(0.2/552.4)
IFNg_Pg	0.9	(0/633.3)	1.6	(0/9.2)	0.7	(0/633.3)	2.1	(0/9.2)	0.6	(0/4.8)	0.4	(0.2/1)	0.5	(0/4.8)	0.4	(0.2/1)
IFNg_Aa	2.9	(0/230.1)	1.1	(0/405.2)	2.9	(0/230.1)	1.1	(0/405.2)	1.0	(0/119.1)	2.1	(0.2/240)	1.0	(0/119.1)	3.2	(0.2/240)
IFNg_Tf	**2.6** ^ #^	**(0.1/633.3)** ^ #^	0.4	(0/9.9)	**2.1** ^*^	**(0.1/633.3)** ^*^	0.4	(0/9.9)	1.6	(0/89.4)	0.3	(0.1/99.5)	0.4	(0/89.4)	0.4	(0.1/99.5)
IFNg_PI	0.8	(0/238.7)	0.2	(0/1.9)	0.8	(0/238.7)	0.3	(0/2.2)	0.7	(0/61.1)	0.2	(0.1/809.9)	0.3	(0/61.1)	0.2	(0.1/809.9)
IFNg_unstim.	0.7	(0/21.9)	0.1	(0/8.6)	0.6	(0/21.9)	0.1	(0/8.6)	0.0	(0/91)	0.1	(0/7)	0.0	(0/91)	0.1	(0.1/0.4)

IL-10_PWM	63.5	(0/3447)	60.3	(0/518.6)	90.3	(0/3447)	55.1	(0/518.6)	76.0	(0/1017)	85.7	(37.4/326)	76.0	(0/1017)	180.7	(37.4/326)
IL-10_P + C	37.2	(0/866.7)	30.4	(0/1732.9)	41.0	(0/866.7)	27.6	(0/1732.9)	125.9	(8.8/497.2)	105.1	(26.3/537.7)	125.9	(8.8/497.2)	105.1	(26.3/537.7)
IL-10_HSP70	5.1	(0/581.3)	1.7	(0/271.7)	5.6	(0/581.3)	3.2	(0/271.7)	9.7	(1.3/250.3)	10.3	(3.9/215.4)	10.4	(1.3/250.3)	4.8	(3.9/215.4)
IL-10_Pg	2.5	(0/164.5)	2.3	(0/81.9)	1.8	(0/164.5)	3.6	(0/81.9)	0.5	(0/45.7)	0.3	(0.1/9)	0.5	(0/45.7)	0.3	(0.1/6.2)
IL-10_Aa	28.8	(0.8/823.6)	25.2	(0.1/589.9)	36.8	(0.8/823.6)	24.0	(0.1/589.9)	26.6	(11.2/303.1)	58.2	(4.4/663.1)	26.6	(4.4/303.1)	92.5	(10.7/663.1)
IL-10_Tf	68.8^†^	(3.6/1510.4)^†^	18.2	(0.1/374.8)	106.9^†^	(9.4/1510.4)^†^	20.6	(0.1/374.8)	**134.2** ^*^	**(4.3/320.1)** ^*^	52.9	(0.6/151.6)	92.6	(0.6/320.1)	63.8	(0.7/151.6)
IL-10_PI	**25.0** ^ #^	**(0/207.2)** ^ #^	2.7	(0/27.6)	**22.7** ^*^	**(0/207.2)** ^*^	3.0	(0/81.9)	9.2	(0/173.6)	3.7	(0.4/29.1)	5.7	(0/173.6)	5.5	(1.1/29.1)
IL-10_unstim.	2.7	(0/14.7)	0.4	(0/7.2)	2.0	(0/14.7)	0.6	(0/7.2)	0.4	(0/11)	2.1	(0.7/6.2)	0.7	(0/11)	2.8	(0.7/6.2)

IL-17_PWM	8.0	(0/2200)	15.0	(5.1/1248.5)	8.5	(0.5/2200)	13.5	(0/1248.5)	47.2	(0.8/2064.1)	127.0	(2.3/947.3)	47.2	(0.8/2064.1)	127.0	(3/947.3)
IL-17_P + C	34.4	(1.1/2200)	62.1	(2.8/2200)	50.2	(1.1/2200)	42.2	(1.5/2200)	239.2	(2.8/2200)	140.4	(0.7/2376.8)	239.2	(0.7/2200)	140.4	(1.1/2376.8)
IL-17_HSP70	6.3	(0/1107.5)	1.2	(0/968.6)	4.0	(0/1107.5)	1.4	(0/968.6)	7.2	(0.1/349.5)	0.3	(0.1/737.9)	7.2	(0.1/349.5)	0.3	(0.1/737.9)
IL-17_Pg	1.1	(0/543.1)	0.4	(0/12.9)	1.0	(0/543.1)	1.2	(0/12.9)	0.1	(0/6.9)	0.3	(0.1/0.6)	0.1	(0/6.9)	0.3	(0.1/0.4)
IL-17_Aa	2.6	(0.8/88.3)	1.1	(0.1/45.1)	2.4	(0.8/88.3)	1.9	(0.1/45.1)	0.3	(0/10.2)	1.2	(0/72.2)	0.4	(0/10.2)	1.2	(0/72.2)
IL-17_Tf	1.6	(0/2064.1)	1.5	(0/22.6)	1.5	(0/2064.1)	2.1	(0/22.6)	0.7	(0/11.6)	0.4	(0/168.9)	0.3	(0/11.6)	1.4	(0/168.9)
IL-17_PI	1.5	(0/62.1)	0.0	(0/6.3)	1.5	(0/62.1)	0.1	(0/6.3)	0.4	(0/10.2)	0.4	(0/2.2)	0.3	(0/10.2)	1.5	(0/2.2)
IL-17_unstim.	1.2	(0/5.1)	0.5	(0/6.3)	1.0	(0/5.1)	0.8	(0/6.3)	0.2	(0/2.8)	0.3	(0/22.7)	0.2	(0/22.7)	0.3	(0/2.5)

IL-1 alfa_PWM	141.3	(0/2400)	160.7	(22/7780)	152.0	(0/2400)	158.7	(22/7780)	231.3	(0/967)	258.7	(112/774.1)	197.1	(0/967)	412.2	(212.2/774.1)
IL-1 alfa_P + C	140.0	(2.3/4701.5)	124.6	(3.6/1643.5)	140.6	(4.3/4701.5)	124.2	(2.3/1643.5)	159.9	(15.4/1153.3)	247.1	(121.9/436.8)	159.9	(15.4/1153.3)	261.1	(242.5/436.8)
IL-1 alfa_HSP70	32.8	(0/1139.8)	13.0	(0/187.4)	52.3	(0/1139.8)	13.0	(0/187.4)	24.1	(6.4/398.9)	**124.0** ^*^	**(43.3/496.4)** ^*^	31.1	(6.4/398.9)	124.0	(43.3/496.4)
IL-1 alfa_Pg	47.5	(4.3/635.4)	44.0	(1.9/339)	47.5	(4.4/635.4)	44.7	(1.9/339)	38.4	(6.8/372.5)	69.9	(53.8/263.5)	44.2	(6.8/372.5)	63.8	(53.8/263.5)
IL-1 alfa_Aa	97.2	(11.2/724.2)	99.3	(2.8/1745.6)	97.2	(13.3/724.2)	110.5	(2.8/1745.6)	129.7	(13/238.7)	**350.3** ^*^	**(108.3/821.5)** ^*^	133.5	(13/238.7)	**405.3** ^*^	**(167.8/821.5)** ^*^
IL-1 alfa_Tf	106.3	(0/450.7)	37.7	(3.3/188.4)	106.3	(0/450.7)	46.7	(3.3/194.9)	121.4	(18.6/720.9)	236.9	(0.2/465.6)	111.5	(18.6/720.9)	355.3	(0.2/465.6)
IL-1 alfa_PI	134.2	(15.9/248.2)	17.0	(1.9/238.7)	133.3	(15.9/248.2)	34.4	(1.9/238.7)	84.4	(11.9/218.5)	242.3	(11.9/346.4)	84.6	(11.9/346.4)	242.3	(11.9/287)
IL-1 alfa_unstim.	17.4	(0/185.5)	6.8	(0/397.3)	**18.8** ^*^	**(0/185.5)** ^*^	9.0	(0/397.3)	20.8	(2.3/120.6)	**140.2** ^*^	**(5.9/308.8)** ^*^	22.5	(2.3/178.1)	140.2	(5.9/308.8)

IL-1 beta_PWM	583.3	(0/2586)	267.1	(0/1597)	**655.9** ^*^	**(0/2586)** ^*^	214.2	(0/1597)	676.4	(2.6/1600)	839.7	(100.9/1700)	676.4	(2.6/1600)	839.7	(285.3/1700)
IL-1 beta_P + C	533.3	(1.3/2650)	278.3	(1.6/1600)	**600.2** ^*^	**(2.6/2650)** ^*^	266.5	(1.3/1600)	695.2	(47.2/1600)	610.5	(168.2/1657)	571.9	(47.2/1600)	733.8	(324.8/1657)
IL-1 beta_HSP70	141.5	(0/1600)	34.7	(0/616.1)	**228.9** ^*^	**(0/1600)** ^*^	34.7	(0/616.1)	93.6	(44.4/1600)	335.9	(183.2/1338.1)	143.4	(44.4/1600)	335.9	(198.5/1338.1)
IL-1 beta_Pg	33.2	(0/851.4)	13.8	(0/600.4)	33.2	(0/851.4)	16.3	(0/600.4)	22.9	(3.7/106)	8.2	(2.4/289.7)	22.9	(2.9/289.7)	8.2	(2.4/189.5)
IL-1 beta_Aa	533.3	(0/4216.5)	482.3	(0/1600)	**533.3** ^*^	**(0/4216.5)** ^*^	405.2	(0/1600)	439.5	(47.7/1271.5)	483.6	(167.5/1531.1)	439.5	(47.7/1271.5)	748.0	(344.3/1531.1)
IL-1 beta_Tf	**516.8** ^*^	**(0/2952)** ^*^	109.6	(0/952.7)	**533.3** ^ #^	**(0/2952)** ^ #^	101.8	(0/952.7)	521.6	(47.7/1600)	448.5	(0/1700)	479.4	(47.7/1600)	558.8	(0/1700)
IL-1 beta_PI	251.0	(0/552.8)	63.8	(0/578.6)	244.7	(0/552.8)	68.7	(0/578.6)	302.9	(145.5/985)	342.0	(44.4/748.2)	302.9	(145.5/985)	390.8	(44.4/748.2)
IL-1 beta_unstim.	**83.5** ^*^	**(0/968.5)** ^*^	21.9	(0/533.3)	**91.8** ^*^	**(0/968.5)** ^*^	16.6	(0/533.3)	66.0	(9.5/621.8)	195.2	(21.5/415.6)	66.0	(9.5/621.8)	247.7	(21.5/415.6)

**(b):** 

Locus	Periodontitis patients								Healthy controls							
	IL-4-590				IL-4VNTR				IL-4-590				IL-4VNTR			
Polymorphism	CC		CT + TT		11		12 + 22		CC		CT + TT		11		12 + 22	
Number of patients	(30)		(16 + 1)		(28)		(17 + 1)		(8)		(5 + 2)		(10)		(5 + 0)	
Cytokines [pg/mL]	Median	(Min/max)	Median	(Min/max)	Median	(Min/max)	Median	(Min/max)	Median	(Min/max)	Median	(Min/max)	Median	(Min/max)	Median	(Min/max)
IL-4_PWM	3.6	(0/163.6)	3.9	(0/17.8)	3.7	(0/163.6)	3.4	(0/17.8)	4.3	(0.6/163.6)	3.6	(0.7/9.9)	4.3	(0.6/163.6)	3.6	(0.7/9.9)
IL-4_P + C	3.7	(0/40.7)	4.4	(0/25.7)	4.0	(0/40.7)	4.0	(0/25.7)	0.9	(0/20.1)	2.2	(0.7/19.9)	0.9	(0/20.1)	2.2	(0.7/11.9)
IL-4_HSP70	0.7	(0/6.5)	0.6	(0/5.5)	0.0	(0/6.5)	1.2	(0/5.5)	1.2	(0/3.1)	1.6	(0.7/2.3)	1.2	(0/3.1)	1.6	(0.7/2.3)
IL-4_Pg	0.5	(0/12.9)	0.1	(0/7.7)	0.1	(0/12.9)	0.1	(0/7.7)	0.7	(0/96.6)	0.7	(0/1.2)	0.7	(0/96.6)	0.7	(0.6/1.2)
IL-4_Aa	0.4	(0/11.2)	0.1	(0/3.7)	0.1	(0/4.7)	0.1	(0/11.2)	1.9	(0.7/113.8)	0.7	(0/4.5)	1.9	(0.7/113.8)	0.7	(0/4.5)
IL-4_Tf	1.8	(0/113.8)	0.8	(0/3.7)	0.1	(0/113.8)	1.6	(0/96.6)	1.2	(0/5.7)	1.2	(0.7/2.6)	1.2	(0/5.7)	1.2	(0.7/2.6)
IL-4_PI	0.7	(0/4)	0.0	(0/3.7)	0.3	(0/4)	0.6	(0/3.7)	0.7	(0/4.1)	0.7	(0/1.9)	0.7	(0/4.1)	0.7	(0/1.9)
IL-4_unstim.	0.0	(0/3.7)	0.0	(0/3.3)	0.0	(0/3.7)	0.0	(0/3.3)	0.9	(0/2.8)	0.7	(0/4.5)	0.9	(0/4.5)	0.7	(0/2.6)

IL-5_PWM	5.7	(0/1882)	3.7	(0/98.9)	8.4	(0/1882)	1.4	(0/98.9)	7.3	(0.6/212.4)	10.6	(1.8/143)	7.3	(0.6/212.4)	10.6	(1.8/112.4)
IL-5_P + C	8.7	(0/230.1)	9.5	(0/375.5)	9.0	(0/230.1)	6.9	(0/375.5)	20.0	(2.3/226.2)	9.2	(1.2/403.2)	20.0	(2.3/403.2)	9.2	(1.2/169.3)
IL-5_HSP70	1.5	(0/64.8)	0.3	(0/61)	1.0	(0/64.8)	0.6	(0/61)	3.8	(0.6/158)	6.0	(1.8/16.9)	4.3	(0.6/158)	5.2	(1.8/16.9)
IL-5_Pg	1.0	(0/322.7)	1.2	(0/8.1)	0.9	(0/322.7)	1.2	(0/8.1)	3.0	(0.6/6)	3.8	(3.2/7.7)	3.5	(0.6/7.7)	3.7	(3.2/6)
IL-5_Aa	1.7	(0/12.5)	1.0	(0/7.9)	1.5	(0/12.5)	1.2	(0/7.9)	3.3	(2.2/5.2)	5.2	(0/6)	3.3	(2.2/6)	5.2	(0/6)
IL-5_Tf	1.9	(0/212.4)	0.6	(0/3.7)	1.8	(0/212.4)	1.2	(0/3.9)	2.0	(1.2/5.2)	2.9	(1.2/6)	2.4	(1.2/6)	2.9	(1.2/6)
IL-5_PI	1.5	(0/28.6)	0.6	(0/3.7)	1.7	(0/28.6)	0.6	(0/3.7)	2.4	(0.6/4.2)	3.4	(0.6/6.9)	2.9	(0.6/4.7)	3.4	(0.6/6.9)
IL-5_unstim.	0.7	(0/8.1)	0.7	(0/7.9)	0.6	(0/8.1)	0.9	(0/7.9)	2.5	(0/6)	3.4	(0/6.9)	2.5	(0/6)	3.4	(1.8/6.9)

IL-6_PWM	3500.0	(0/5960)	3500.0	(0/4699)	3500.0	(5.4/5960)	3500.0	(0/4699)	2143.1	(0/4824.3)	3281.8	(130.5/5200)	2143.1	(0/5200)	3281.8	(1186.1/4272.5)
IL-6_P + C	3196.5	(0/6457)	2280.7	(44.7/5528.5)	3500.0	(9.3/6457)	2267.5	(0/5528.5)	2876.5	(211.1/4900)	1593.1	(123.2/5193.4)	2876.5	(211.1/5193.4)	1593.1	(123.2/4931.7)
IL-6_HSP70	773.9	(3.3/4207)	141.4	(2.7/5833.5)	1143.1	(3.3/4207)	51.9	(2.7/5833.5)	888.5	(165.6/3500)	2040.7	(167.5/4673.4)	888.5	(165.6/4673.4)	2040.7	(167.5/3111.3)
IL-6_Pg	19.8	(0/4191.6)	10.6	(0.4/3577.2)	19.8	(0/4191.6)	11.5	(0.4/3577.2)	13.8	(0/791)	6.5	(0.3/2438.4)	13.8	(0/2438.4)	6.5	(0.3/1675.2)
IL-6_Aa	3500.0	(27.5/6667)	3500.0	(71.9/7362.5)	3500.0	(839/6667)	3500.0	(27.5/7362.5)	3500.0	(108/4686)	2076.7	(258.5/3600)	3366.9	(108/4686)	2076.7	(1802.6/3600)
IL-6_Tf	3500.0	(25.1/6760)	1035.3	(1.4/4916)	**3500.0** ^*^	**(108/6760)** ^*^	913.9	(1.4/4916)	3457.2	(33.9/4203.3)	2037.2	(0.2/3500)	3449.9	(33.9/4203.3)	2037.2	(0.2/3500)
IL-6_PI	**1052.6** ^*^	**(336.1/4032)** ^*^	350.9	(0.7/3521.6)	**1102.1** ^*^	**(336.1/4032)** ^*^	585.5	(0.7/3521.6)	3011.5	(6.2/4686)	324.3	(87.5/1937.7)	2387.8	(6.2/4686)	324.3	(87.5/1337)
IL-6_unstim.	214.8	(2/3200.5)	20.0	(1/2051.9)	227.2^*^	(2/3200.5)^*^	8.0	(1/2051.9)	468.4	(33.9/1796.2)	558.5	(14.2/2046.3)	418.9	(14.2/1796.2)	601.9	(25.2/2046.3)

TNF alfa_PWM	1085.9	(2.8/3701)	1002.3	(0/3880)	1250.1	(3.5/3701)	963.4	(0/3880)	1506.6	(3.5/2800)	1209.1	(85.1/2800)	1494.2	(3.5/2800)	1209.1	(85.1/2800)
TNF alfa_P + C	1071.5	(0/3871)	450.2	(74/2800)	1127.2	(7.3/3871)	442.1	(0/2800)	1579.4	(35.6/2800)	1531.3	(22.9/2800)	1596.6	(35.6/2800)	1531.3	(22.9/2800)
TNF alfa_HSP70	32.1	(2.6/3494.6)	11.6	(2.3/2800)	92.6	(2.6/3494.6)	10.6	(2.3/2800)	63.4	(9.6/1249.1)	152.2	(11.8/1656.7)	87.6	(9.6/1656.7)	152.2	(11.8/432)
TNF alfa_Pg	20.6	(0.5/612.2)	13.6	(1.9/576.6)	21.1	(0.5/612.2)	13.3	(1.9/576.6)	2.1	(0.3/54.6)	0.8	(0.2/146.3)	2.1	(0.2/146.3)	0.8	(0.4/51.8)
TNF alfa_Aa	407.1	(10.7/4760)	462.2	(3.7/3880)	510.8	(10.7/4760)	441.8	(3.7/3880)	933.0	(104.4/3569.7)	527.6	(51.9/4347.3)	933.0	(51.9/3569.7)	527.6	(235.7/4347.3)
TNF alfa_Tf	650.9	(4.2/2845.4)	179.7	(2.1/2393.9)	**687.5** ^*^	**(104.4/2845.4)** ^*^	147.2	(2.1/2393.9)	899.8	(8.5/1813.5)	387.6	(0.1/965.3)	886.2	(8.5/1813.5)	387.6	(0.1/965.3)
TNF alfa_PI	74.0	(8.3/1448.7)	39.1	(1.2/182.6)	73.4	(8.3/1448.7)	55.0	(1.2/297.7)	80.5	(22.2/130.7)	38.2	(9.5/223)	73.4	(10.4/130.7)	38.2	(9.5/223)
TNF alfa_unstim.	14.0	(1.8/2336.7)	6.6	(1.7/91.8)	16.7	(1.8/2336.7)	4.8	(1.7/91.8)	7.0	(2.1/52.1)	21.1	(3.5/112.1)	10.5	(2.1/52.1)	18.7	(3.5/112.1)

VEGF_PWM	13.7	(3.4/66.9)	5.5	(1.5/94.4)	14.4	(3.4/66.9)	5.1	(1.5/94.4)	17.1	(0.2/83.4)	4.9	(0.1/33.1)	11.0	(0.2/83.4)	11.9	(0.1/33.1)
VEGF_P + C	21.7	(4.7/96.1)	9.4	(1.6/52.6)	27.2	(4.7/96.1)	9.3	(1.6/52.6)	12.3	(0/20.6)	5.2	(0.2/17.2)	9.6	(0/20.6)	5.2	(0.2/17.2)
VEGF_HSP70	32.8	(5.8/101.1)	10.6	(1.1/45.3)	35.5	(5.8/101.1)	16.0	(1.1/45.3)	54.0	(3/121.7)	14.4	(1.5/91.9)	42.7	(3/121.7)	15.7	(1.5/91.9)
VEGF_Pg	17.9	(0.5/48.1)	9.4	(0.8/63.6)	17.4	(0.5/48.1)	15.3	(0.8/63.6)	2.9	(0.3/33.7)	5.5	(0.1/30)	5.1	(0.3/33.7)	2.1	(0.1/30)
VEGF_Aa	27.4	(8.8/119.1)	27.7	(3.5/57.8)	27.0	(8.8/119.1)	29.5	(3.5/57.8)	47.8	(4.1/73.7)	27.8	(7.6/36.8)	23.9	(4.1/73.7)	30.4	(7.6/36.8)
VEGF_Tf	18.4	(4.1/89.4)	17.0	(4/90.1)	17.2	(4.1/89.4)	19.0	(4/90.1)	18.9	(3.6/126.8)	11.1	(0.1/27.3)	10.6	(3/126.8)	13.9	(0.1/27.3)
VEGF_PI	30.2	(7.1/94.4)	15.2	(4.6/72.8)	29.1	(7.1/94.4)	22.6	(4.6/72.8)	30.2	(20/97.8)	15.2	(0.1/49.9)	29.4	(10/97.8)	15.2	(0.1/49.9)
VEGF_unstim.	24.9	(0.9/91)	22.8	(4.5/135.5)	29.6	(0.9/91)	21.9	(4.5/135.5)	39.8	(15.3/79.4)	10.2	(6.4/42.4)	35.6	(9.5/79.4)	10.2	(6.4/42.4)

^*^ , ^#^, and ^†^ denote significantly higher levels of cytokines for *P* < 0.05, *P* < 0.01, and *P* < 0.001 (resp.), how they were found by Kruskal-Wallis tests in application to couple locus in table header (the minor homozygous locus was connected to a heterogeneous locus for the formal increase the statistical significance of results).
